# 

**Published:** 1890-08

**Authors:** 


### CONTENTS:

Article I.—Dental Education. By C. Stoddard Smith, D.D.S. 145
" II.—Calcification. By Sidney Spokes, M.R.C.S., L.D.S. 155
" III.—Tin and Gold in Combination for Filling Teeth. By Professor W. D. Miller. - - - - - 162
" IV.—Tannic Acid: Its Internal Administration for Hemorrhage After Tooth Extraction. By D. W. L. Roberts. - - - - - - - 167
" V.—The First Permanent Molar. By J. F. Colyer, M.R.C.S., L.R.C.P., L.D.S. - - - - 171
" VI.—Immediate Root Fillings. By Dr. M. S. Merchant. 174
" VII.—Hypnotism in Dentistry. - - - - 177

### COMMUNICATIONS.

Missouri State Dental Association. - - - - - 179
National Association of Dental Examiners. - - - - 182
National Association of Dental Faculties. - - - - 186

### EDITORIAL.

Germs in a Baby's Jaw. - - - - - - - 189

### BIBLIOGRAPHICAL.

A New Medical Dictionary. - - - - - - 190
A New Dental Journal. - - - - - - 191
A Change of Editors. - - - - - - - 191
Catching's Compendium of Practical Dentistry. - - - 192
The Student's Manual and Hand-Book for the Laboratory. - 192

